# A Route to Translate a Silk-Based Medical Device from Lab to Clinic: The Silk Biomaterials Srl Experience

**DOI:** 10.3390/insects13020212

**Published:** 2022-02-21

**Authors:** Giulia Alessandra Bassani, Valentina Vincoli, Marco Biagiotti, Elisa Valsecchi, Marta Virginia Zucca, Claudia Clavelli, Antonio Alessandrino, Giuliano Freddi

**Affiliations:** Silk Biomaterials Srl, 22074 Lomazzo, Como, Italy; giulia.bassani@outlook.it (G.A.B.); valentina@silkbiomaterials.com (V.V.); marco@silkbiomaterials.com (M.B.); elisa@silkbiomaterials.com (E.V.); marta@silkbiomaterials.com (M.V.Z.); claudia@silkbiomaterials.com (C.C.); antonio@silkbiomaterials.com (A.A.)

**Keywords:** silk fibroin, SILKBridge^®^ nerve conduit, performance testing, biocompatibility, toxicology, regulatory requirements, quality requirements, control manufacturing

## Abstract

**Simple Summary:**

Silk has always been a source of inspiration for textile designers to create very disruptive fashion products. More recently, silk has stimulated the creativity of scientists, as it emerged as a promising biomaterial for the development of next generation medical devices. Although silk has been clinically used as a suture for decades, only the development of novel processing approaches paved the way for the production of a plurality of regenerated silk-based materials, i.e., films, hydrogels, sponges, powder, nano- and microparticles, nanofibers, etc., which have been recognized as scaffolds-of-choice in different medical applications. However, the translation of research achievements into medical products used in clinical settings is complex from manufacturing, quality, and regulatory perspectives. The aim of this paper is to disclose how the clinical translation route works using, as a case study, a silk-based medical device recently developed by the Italian start up Silk Biomaterials srl. The results reported here will cover some fundamental aspects of the regulatory and quality path, from the demonstration of the robustness of the manufacturing process up to the evaluation of the biocompatibility, and of the functional performance of the device.

**Abstract:**

The medical device is a nerve conduit entirely made of *Bombyx mori* silk fibroin. It is a tubular scaffold used for repairing peripheral nerve gaps, whose function is to protect the severed nerves and to favor their natural healing process. As any implantable medical device, the conduit must perform its function without causing adverse effects to the patient, meaning that it must be compliant with a range of regulations aimed at evaluating the risks related to the constituent materials and the manufacturing process, the toxicological impact of the processing aids, the biological safety, the functional performance, and the ability to sustain tissue regeneration processes. An exhaustive on-bench testing plan has been performed for the determination of the morphological, geometrical, physical, structural, and mechanical properties. For the toxicological analysis, the device was extracted with solvent and the number of leachable substances was determined by suitable chromatographic techniques. The biological safety was assessed by means of a set of tests, including cytotoxicity, delayed hypersensitivity, intracutaneous reactivity, pyrogen test, LAL (Limulus Amebocyte Lysate) test, acute systemic toxicity, and genotoxicity. Overall, the accumulated results demonstrated the suitability of the device for the intended use and supported the starting of a first-in-human clinical trial.

## 1. Introduction

Silk is a fibrous protein secreted by leaving organisms (insects, spiders, etc.) for housing, protection, or predation purposes. It is synthesized into the cells of specialized glands, accumulated into the lumen, and finally extruded to make extracorporeal filamentous structures (nets, cocoons, nests, etc.) [1,2]. The silks produced by *Lepidoptera* (*Bombyx*, *Antheraea*, *Phylosamia* genus) are an important source of starting materials for the textile industry [3]. The so-called domesticated or mulberry silk, obtained from rearing *Bombyx mori* silkworms and unraveling their cocoons, has been used for thousands of years to produce precious fabrics highly appreciated for their lustrous appearance, softness, and elegance. Silk production represents less than 0.1% of the global fiber market (estimated at about 110 million tons in the year 2020; source: https://textileexchange.org, accessed on 15 November 2021) but is still a significant billion-dollar industry because silk is the leading fiber in fashion design (https://fashionunited.com, accessed on 15 November 2021).

The first documented medical use of silk dates back to about 2200 years, when Claudius Galenus proposed to use silk as suture thread [4]. Other similar applications appeared throughout the centuries, but only in the 20th century have silk sutures become commercially available for routine clinical use. A key step for the development of a wider range of medical applications has been the dissolution of native silk fibroin fibers and the obtainment of a liquid feedstock from which different regenerated silk-based materials such as films, hydrogels, porous scaffolds, nanofibers, particles, etc., could be prepared [5]. Of the two main components of silk, fibroin and sericin, fibroin is the material that has attracted the most attention, although sericin has recently been carving out a significant space in the biomaterial field [6]. However, every time silk is mentioned in this article, reference will be made solely to the fibroin component.

From the 1980s onwards, the scientific community’s interest in silk as a biomaterial has dramatically increased. Just to give an idea of the development of scientific interest in this area, a quick search in the Scopus database using “silk fibroin” as keyword returned about 50 papers published in the year 2000 and more than 800 in 2020, with an annual increase trend that is expected to continue in the future. While Japanese, European, and American research teams dominated the scientific production about silk at the beginning of the 21st century, the current panorama of the most prolific countries is led by China, whose scientific production is now three-times larger than that of America. It seems, therefore, that the millennial history of silk, which began in China and spread throughout the world via the Silk Road, has made a U-turn to return to where it originated, coupling the more traditional Chinese monopoly in silk production to the newly acquired scientific leadership.

The range of biomedical applications targeted by silk-based scaffolds is very wide, spanning from implantable devices for engineering and/or regeneration of soft and hard tissues to the development of tissue models, carriers for drug release, diagnostic devices, etc. [4,7,8]. The reason for such a wide interest in silk lies in its intrinsic properties, including biocompatibility, controllable degradability, absence of toxicity of degradation products, structural stability, mechanical performance, wide choice of manufacturing options which makes it possible to design scaffolds with desirable features for specific applications [5]. Different silk-based products have reached the pre-clinical stage and have been tested in small and large animal models, and in clinical trials with promising results [4]. Even if the number of silk-based devices approved for clinical use is still very low, the number of companies involved in the development of silk-based medical products has been increasing over the last decade; thus, it is likely that, quite soon, many of them will undertake the regulatory path that leads to the approval of devices for use in the clinical setting [4,9].

The translational process that allows a silk-based material to go through the regulatory pathway that transforms the initial idea and the starting material into a product to be used in the therapeutic field is particularly long and complex. Although there might be differences depending on the different rules put in place by European (CE mark) or American (Food and Drug Administration-FDA approval) regulatory agencies, many basic steps are common. These include the acquisition of raw materials in accordance with well-established quality assurance programs, the rigorous control of the manufacturing process that must be robust and compliant in terms of product specification, the implementation and execution of on-bench and in vitro testing programs, the thorough biocompatibility evaluation of the product according to guidelines such as ISO 10993-1 (International Organization for Standardization), the execution of relevant in vivo functional tests using appropriate animal models, the access to clinical trials to evaluate safety and performance of the device and the submission of the dossier to a regulatory body for device approval [9].

The aim of this paper is to disclose how the clinical translation route works using the experience of the Italian start up Silk Biomaterials srl in the development of a silk-based medical device, the SILKBridge^®^ nerve conduit, as case study. The device is designed for the surgical repair of peripheral nerve discontinuities to support the regeneration of injured nerves [10]. It is intended to act as a bridge to guide and structurally support axonal growth across the gap during the healing process. The conduit is entirely made of silk fibroin obtained from *Bombyx mori* silkworms. During the manufacturing process, sericin is completely removed by degumming to leave pure silk fibroin. The conduit has a tubular structure with a three-layered wall comprising an intermediate textile layer intimately and firmly coupled with two electrospun layers, one in the inner and the other in the outer parts of the device wall [10]. This design allowed for optimizing both mechanical and biological properties, because the electrospun layers have biomimetic characteristics that enhance cell’s adhesion and integration with the surrounding tissues, while the textile layer was designed to provide the required mechanical resistance during implantation (suture retention strength) and in vivo functioning (compression strength) [11]. The conduit displayed a slow degradation rate both in vitro [12] and in vivo [11]. As degradation occurred, the load bearing responsibility was transferred to the new tissue ingrowth such that mechanical integrity was maintained at the implantation site and a complete morphological and functional recovery of the transected nerve was achieved [11].

The results reported here complement those already published [10,11,12] about the synergic efficacy of the use of silk as biomaterial and of the design of a three-layered wall architecture for manufacturing the nerve conduit; moreover, they specifically tackle the following issues of the regulatory process: demonstration of the robustness of the production process through the evaluation of the functional parameters of the device defined in the design phase, verification of potential sources of risk that may emerge from the chemicals used for production through toxicological analysis, evaluation of biological safety as required by regulations in force for medical devices and start of the first phase of clinical trials.

## 2. Materials and Methods

### 2.1. Starting Materials and Device Manufacturing

The wall of the tubular device consists of three layers: two electrospun layers (ES, inner and outer) and an intermediate textile layer (TEX) [10]. Two different raw materials were used to produce the device: (i) a degummed silk yarn (organzine, 40 dtex), with which a tubular braid was manufactured by warp needle braiding technology; the braid forms the intermediate TEX layer; (ii) silk cocoons, which were degummed in an autoclave to remove sericin; the pure silk fibroin fibers were used as starting material to produce the inner and outer ES layers. Both silk cocoons and yarn were acquired from external suppliers, in compliance with traceability requirement. The incoming raw materials entered the device’s manufacturing cycle after having passed control tests aimed at addressing quality and purity requirements.

The manufacturing process has been described in detail elsewhere [10]. Briefly, the TEX and ES layers comprising the wall of the device were coupled during electrospinning according to a patented process [13]. Two ES layers were assembled onto the inner and outer faces of a TEX textile braid. Coupling of the TEX layer with the two ES layers was made by means of two different welding media: (i) a solution of ionic liquid (1-ethyl-3-methylimidazolium acetate; EMIMAc; #51053, Sigma-Aldrich, Merk Life Science S.r.l., Milano, Italy) in water (EMIMAc/water 80/20% *v*/*v*); (ii) a solution of 15% *w*/*w* SF in EMIMAc. After electrospinning, the three-layered tubular structure was consolidated by immersion in 80% *v*/*v* ethanol for 30 min at room temperature, followed by overnight washing with distilled water and drying. Finally, the tubular structure was purified by extraction with ethanol (EtOH) in a microwave assisted extractor, washed with ultrapure water, dried, packaged in double pouches under laminar flow cabinet, and, finally, sterilized with ethylene oxide.

### 2.2. On-Bench Testing

#### 2.2.1. Relaxed Internal Diameter

The cross-sections of the final finished devices were mounted on aluminum stubs and sputter coated with Au/Pd (Desk V, Denton Vacuum, LLC, Moorestown, NJ, USA). A Zeiss EVO MA10 scanning electron microscope (SEM) operating at 10 kV acceleration voltage, 100 μA beam current, and 35 mm working distance was used to acquire the image of the tubular cross-section of the device. The internal diameter was determined from the SEM image using the SEM software measuring tools.

#### 2.2.2. Wall Thickness

Wall thickness was measured according to the ISO 7198:2016 standard method. The tubular device was cut longitudinally, flattened, and measured with a thickness tester MarCator 1075R (Mahr) equipped with a constant load thickness gauge of 0.3 cm^2^ foot area that exerts a pressure of 1 kPa.

#### 2.2.3. Linear Density (Mass Per Unit Length)

The length (L) of the sample was measured with a digital Vernier caliper (Metrica, Brighton, East Sussex, UK; Carbon Fiber Composite Digital Caliper), with 0.01 cm resolution. The mass (M) of the samples was measured with an analytical balance (Mettler Toledo, Milano, Italy, XRS105 Dual Range), with 0.01 mg resolution. The linear density (mass per unit length) of the sample, expressed in mg/cm, was calculated from the M/L ratio.

#### 2.2.4. Porosity

Porosity (%P) was determined according to ISO 7198:2016, using the gravimetric test procedure, according to the following formula:

%P = 100 × [1 − (M/V*ρ*)]

where:

- M is the mass of the device (see: Section 2.2.3);
- V is the volume of the device, calculated from the values of wall thickness (see: Section 2.2.2) and relaxed internal diameter (see: Section 2.2.1);
- *ρ* is the density of silk fibroin (1.35 g/cm^3^) [14].

#### 2.2.5. Degree of Crystallinity

Attenuated total reflectance Fourier transform infrared spectroscopy (ATR-FTIR) was used to determine the degree of crystallinity. ATR-FTIR spectra were acquired with an ALPHA FTIR spectrometer (Bruker) equipped with an ATR platinum diamond accessory, at a resolution of 4 cm^−1^, in the infrared range of 4000–400 cm^−1^. The tubular specimen was cut longitudinally and the surface of the inner electrospun layer was pressed against the ATR crystal. The spectra recorded were corrected with a linear baseline and normalized to the CH_2_ bending peak at about 1445 cm^−1^. The crystallinity index was calculated by ratioing the intensity of the Amide III bands at 1260 cm^−1^ and 1230 cm^−1^ (CI = A_1260_/A_1230_) [15].

#### 2.2.6. Thermal Properties

Thermal properties were determined by differential scanning calorimetry (DSC) using a calorimeter DSC 3500 Sirius (Netzsch, Selb, Germany). Samples (3–5 mg) were sealed in aluminum pans and subjected to a heating cycle from 50 °C to 400 °C, at a heating rate of 10 °C/min, under an N_2_ atmosphere (flow rate: 20 mL/min). The following parameters were determined from the DSC curves: peak temperature of the endotherms associated with melting/degradation of the ES (T_ES_) and TEX (T_TEX_) layers; cumulative enthalpy (DH_ES/TEX_) of the ES and TEX endotherms; ES:TEX weight ratio, calculated from the relative intensity of the respective DSC endotherms, using the DH values of −402 J/g and −307 J/g for the pure TEX and ES layers, respectively, to normalize the contribution of the two materials.

#### 2.2.7. Compression Strength

Compression tests were performed under wet conditions, using an all-electric dynamic test instrument ElectroPuls E3000 (Instron, Norwood, MA, USA), equipped with a load cell of 10 N, a thermostatic bath (BioPuls), and appropriate grips submersed in water at 37 °C [10]. The sample of 10 mm length was mounted between the upper and lower plates and tested at a crosshead speed of 1.0 mm/min, with a preload of 0.1 N. The sample was conditioned in the thermostatic bath for 5 min before starting the test. The force was applied perpendicular to the longitudinal axis of the tubular sample. Compressive load values were recorded at strains of 20% and 40%. The modulus was calculated from the slope of the compression curve in the 0–40% load range.

#### 2.2.8. Suture Retention Strength

The suture retention strength is the force necessary to pull a suture from the device. The ElectroPuls E3000 (Instron) tester equipped with a load cell of 10 N was used. The conduit was cut normal to the long axis and a suture was inserted 2 mm from the end of the sample through the wall to form a half loop. The sample was clamped in the lower fixed grip and the suture thread in the upper moving grip, which was pulled at the rate of 50 mm/min. The test was performed in the wet state with the sample submersed at 37 °C. The sample was conditioned in the thermostatic bath for 5 min before starting the test. The force required to pull the suture through the device was recorded.

#### 2.2.9. Tensile Properties

Breaking load and elongation at break in uniaxial tensile test were determined under submersed conditions (in water at 37 °C), with the ElectroPuls E3000 (Instron) tester, equipped with a 250 N load cell. The test was performed in accordance with the provisions of ISO 7198:2016. The sample was conditioned in the thermostatic bath for 5 min before starting the test. The gauge length was 30 mm; a preload of 0.5 N was applied; the tests were run at 30 mm/min crossbar rate.

#### 2.2.10. Statistical Analysis

Data from different production batches were compared using the one-way ANOVA statistical test to confirm the statistical equivalence between different production lots. Statistical analyses were conducted using Minitab statistical software.

### 2.3. Chemical Analysis

#### 2.3.1. Determination of Bromide Ion

The sample was weighed into a crucible and reduced to ash. Ashes were recovered with water and the solution was filtered with a 0.45 µm filter before analysis by ion chromatography, with a Dionex™ ICS-4000 Integrated Capillary HPIC™ System, equipped with a Dionex IonPac AS11-HC-4 mm column, using a KOH solution as mobile phase in the gradient mode (0.02–80 mM in 15 min), at a flow rate of 15 mL/min. The injection volume was 0.4 mL; detection was made by a conductivity and QD charge detector. The results were expressed in mg/kg.

#### 2.3.2. Determination of Lithium Ion

The sample was digested in a microwave oven (Anton Paar Multiwave 3000) with an acid mixture containing 4 mL HNO_3_/2 mL H_2_O_2_/0.25 mL HF, with a power ramp from 0 to 1200 W in 25 min, a 15 min hold step at 1200 W and a cooling step for 20 min. The analysis was performed by ICP-MS (inductively coupled plasma—mass spectrometry) employing a cross flow nebulizer with a Scott spray chamber (ICP-MS Perkin Elmer SCIEX mod ELAN 9000, autosampler AS90, Waltham, MA, USA). The element was quantified with an external calibration (AccuStandard ICP-MS-CAL2-1 multielement, New Haven, CT, USA), employing Germanium as the internal standard to compensate plasma fluctuations. The results were expressed in mg/kg.

#### 2.3.3. Determination of Methyl and Ethyl Alcohols

The analysis was performed by head-space gas chromatography/mass spectrometry (HS-GC/MS) with a Shimadzu, mod. 2010 Plus, equipped with a MS detector QP2010 Ultra and an autosampler Shimadzu AOC 5000 Plus. Head space conditions were as follows: temperature 80 °C, time 60 min, injection volume 500 μL. A capillary column Agilent HP5-ms (30 m length, 0.25 mm inner diameter, 0.25 μm film thickness) was used for the separation. Other analytical conditions were as follows: carrier gas Helium at 1 mL/min; incubation temperature 80 °C; incubation time 60 min; splitless injector temperature 200 °C; temperature program hold 40 °C for 3 min—from 40 °C to 260 °C at 40 °C/min; injection volume 500 μL; transfer line temperature 280 °C; source temperature 200 °C; E 70 eV; SIM acquisition mode. Target ions were *m*/*z* 32 methyl alcohol (qualifier ion 31), *m*/*z* 31 ethyl alcohol (qualifier ion 45), and *m*/*z* 31 for 1-propanol (internal standard). The results were expressed in mg/kg.

#### 2.3.4. Determination of 1-ethyl-3-methyl Imidazolium Acetate

The samples were finely chopped and extracted with methanol in an ultrasonic bath. The supernatant was filtered on a 0.22 μm PVDF syringe filter, dried, and recovered with 300 μL of eluent A. The analysis was performed by ultra high-pressure liquid chromatography-ion trap-mass spectrometry (UHPLC-IT-LTQ-MS/MS, FinniganMAT), with an Alltima HP C18 (3 μm, 150 mm × 2.0 mm) column, in the gradient mode (Eluent A: H_2_O + 0.1% CH_3_COOH; Eluent B: CH_3_OH + 0.1% CH_3_COOH; from 90% A to 100% B in 17 min), at a flow rate of 0.2 mL/min. The injection volume was 10 μL, the operating mode of the MS detector H-ESI(+), and the mass range *m*/*z* 50–300 uma. The results were expressed in mg/kg.

#### 2.3.5. Determination of Formic Acid

The analysis was performed by head-space gas chromatography/mass spectrometry (HS-GC/MS), by using the same apparatus described in paragraph 2.3.3. for the determination of alcohols. Analytical conditions were the same, with exception of incubation time (60 min) and injection volume (500 μL). Target ions were formic acid (*m*/*z* 46) and acetic acid (*m*/*z* 60; internal standard). The results were expressed in mg/kg.

### 2.4. Biological Safety Evaluation

The ISO 10993 standard was used as benchmark for the biological safety evaluation of the nerve conduit. The following tests were performed: cytotoxicity (ISO 10993-5:2009); delayed hypersensitivity (ISO 10993-10:2010); irritation/intracutaneous reactivity (ISO 10993-10:2010); pyrogenicity (ISO 10993-11:2017; US Pharmacopoeia-USP 40<151>, and European Pharmacopoeia-EU 8.0 §2.6.8.); LAL test (USP <85>; USP <161>; EU par. 2.6.14 e par. 5.1.10; AAMI ST72); acute systemic toxicity (ISO 10993-11:2017); genotoxicity (ISO 10993-3:2014). Tests were carried out at Eurofins Biolab S.r.l. test facility (Good Laboratory Practice-GLP cert. n. 2017/16), Vimodrone (Milan), Italy.

## 3. Results

### 3.1. On-Bench Testing and Property/Performance Evaluation

Sources of possible variability may emerge at different points of the manufacturing process of a medical device and must be carefully monitored to avoid any negative impact on the properties and performance of the final device. Therefore, the design controls requirements according to USA (FDA, 21 CFR 820.30) and EU (MDR, 2017/745) directives were put in place for the design and manufacturing of the SILKBridge^®^ nerve conduit, and an on-bench testing plan has been specifically developed for an in-depth property/performance evaluation. Three test categories, each one addressing specific functional endpoint, were defined. In particular:

1. the morphological and geometrical characteristics, which may affect the usability of the device during surgery, the performance at the site of implantation, and the biological response of surrounding tissues;
2. the physical and structural characteristics, which may impact the biological interaction with surrounding tissues and the rate and extent of degradation after implantation (the device is entirely made from a biodegradable natural protein polymer);
3. the mechanical characteristics, which must achieve a level high enough to withstand the mechanical stresses caused by suturing during implantation, as well as tensile, compression, and bending stresses caused by the surrounding tissues during in vivo functioning.

**Figure 1:**
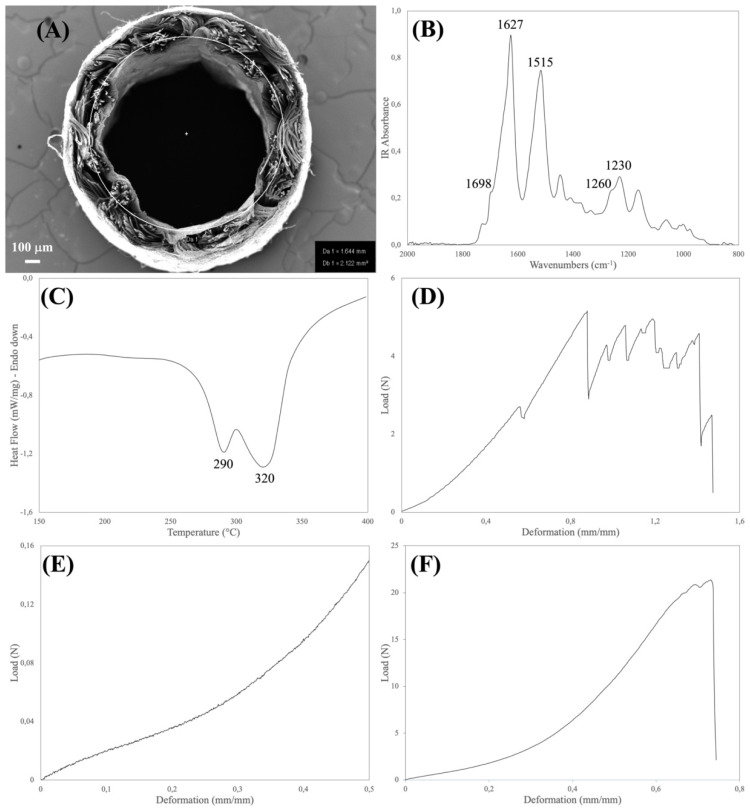
(**A**). SEM cross-section of the SILKBridge^®^ nerve conduit showing the two inner and outer electrospun layers that enclose the intermediate textile layer. (**B**). ATR-FTIR spectrum of the inner electrospun layer in the 2000–800 cm^−1^ range showing the Amide bands characteristic of silk fibroin: Amide I, peak at 1627 cm^−1^ and shoulder at 1698 cm^−1^; Amide II: peak at 1515 cm^−1^; Amide III: peak at 1230 cm^−1^ and shoulder at 1260 cm^−1^. The Degree of crystallinity is calculated from the I_1260_/I_1230_ intensity ratio. (**C**). DSC thermograms of the device in the 150–400 °C temperature range. Peak at 290 °C: thermal degradation of the electrospun component. Peak at 320 °C: thermal degradation of the textile component. (**D**). Typical load/deformation curve of the suture retention test. The series of peaks recorded before ultimate failure represent the breakage of the yarns forming the textile layer. (**E**). Typical load/deformation curve of the compression strength test. (**F**). Typical load/deformation curve of the device analyzed in the uniaxial tensile mode.

At the end of the manufacturing process, each device was visually inspected, cut to its final length (30 mm), and packaged for sterilization. The portions of the device exceeding the length of 30 mm were collected and analyzed for the determination of the morphological, geometrical, physical, and structural properties. This means that each device has undergone testing for the characteristics listed under points 1 and 2. On the other hand, the mechanical properties listed under point 3 were analyzed by sampling (1 device every 50 devices produced) because the tests require an entire device to be executed and are destructive. Figure 1 shows some representative experimental data for the three test categories analyzed on the finished device.

Table 1, Table 2 and Table 3 list the testing results obtained from the analysis of samples representative of three different production batches. For each batch, five samples were analyzed, and the results were reported as mean ± standard deviation. No statistically significant differences were observed among the production batches for each measured characteristic, indicating that the manufacturing process is under control and that the devices will perform as expected.

### 3.2. Chemical Analysis and Toxicological Evaluation

For the manufacturing of medical devices, chemicals could be used as processing aids. The safety profile and the toxicological risks related to their use must be assessed. In the case of SILKBridge^®^, the processing aids used during manufacturing are: (i) methanol (MeOH), to clean starting materials; (ii) ethanol (EtOH), to consolidate the electrospun layers by solvent-induced random coil → β-sheet conformational transition and to clean the final device; (iii) lithium bromide (LiBr), to dissolve degummed cocoon silk fibroin fibers to produce SF films as a preparatory step of the electrospinning process; (iv) formic acid (FA), to dissolve SF films and prepare the electrospinning dope; (v) 1-ethyl-3-methyl imidazolium acetate (EMIMAc), the welding agent used to couple the TEX and ES layers. In terms of hazard classification (Regulation EC No 1272/2008), all these substances share an acute toxicity designation (oral, dermal, or by inhalation); some may cause eye/skin irritation (LiBr, EtOH, EMIMAc); EtOH and MeOH may show specific target organ toxicity, but none of them are reported as carcinogenic, mutagenic, toxic to reproduction or with endocrine-disrupting activity. Despite the thorough cleaning procedures adopted, residues of these chemicals may remain on the device and leach in the surrounding tissues during in vivo implantation. This might impact on the biocompatibility of the device and interfere with the smooth progress of tissue regeneration.

To evaluate the amount of residual processing aids that may remain in the final device, sterilized samples of nerve conduits were extracted, and the extracts were analyzed (Table 4, line 1). These results represent the starting point to perform the toxicological evaluation following the provisions of ISO 10993-17. As a first step, the concentration of each chemical expressed in mg/kg was transformed in μg/device to obtain the total amount of each compound released by one device (line 2). For this calculation, an average weight of 25 mg/device was considered. Then, assuming a worst-case scenario of multiple devices implanted at the same time in one patient (in this case: 10 devices), the value was multiplied by 10 to calculate the potential patient daily intake (PPDI, expressed in μg/day) (line 3), i.e., the total amount of chemical to which the patient is potentially exposed during the lifetime. A further worst case assumption was that the total amount of the chemical expressed by the PDDI value is released just in one day.

For each compound, the toxicological data were retrieved from specialized databases (TOXNET Databases, Pubmed, Pubchem, ECHA—European Chemical Agency, FDA—Food and Drug Administration, etc.). They allowed setting the values of tolerable exposure (TE, expressed in μg/day), i.e., the amount of chemical agent that doesn’t pose concerns for human health (line 4). Since no relevant toxicological data were available for EMIMAc, the TE could not be calculated as recommended by ISO 10993-17. Instead, the value of TTC (threshold of toxicological concern) was estimated according to ISO/TS 21726. It is important to note that the TTC value assigned to EMIMAc (10 μg/day) derives from an extremely conservative and protective classification based on individual mutagenic impurities in pharmaceuticals and is presumed to be protective for both the potential carcinogenic and non-cancer effects that would occur following patient exposure to the medical device. Finally, the TE/PPDI ratio was calculated to obtain the margin of safety (MoS, line 5). For values of MoS ≤ 1, the compound is considered to pose a toxicological concern. For values of MoS > 1, the compound does not raise risks for human health. We adopted a more conservative approach, setting a threshold for MoS ≥ 10. As can be observed in Table 4, despite the worst-case assumptions made at different levels of the toxicological evaluation, i.e., total amount released in one day, multiple devices, MoS threshold higher than recommended by the reference standard, none of the compounds tested can be held responsible for causing toxicological concerns.

### 3.3. Biological Safety Evaluation

The ISO 10993-Part 1 provides a guidance for the assessment of medical devices according to the nature and duration of their contact with human tissues, and the other parts of the standard give indications on how to perform the tests required. SILKBridge^®^ is categorized as an implant device in long term contact with tissue/bone (>30 days). Based on this categorization, as well as on the analysis of the risks associated with the constituent materials, their source, the manufacturing process, and the morphological, physical, chemical, and mechanical characteristics, the endpoints of the biological safety evaluation were established according to the whole ISO 10993 standard. Sterilized devices were then tested for each biological endpoint; the results are summarized in Table 5.

## 4. Discussion

The development of an implantable medical device as the SILKBridge^®^ nerve conduit must undergo a comprehensive series of control tests before being released for use to ensure that the manufacturing process is under control and that the device performs as expected. The on-bench testing framework complies with the design controls requirements set out according to FDA and EU/MDR regulations, and comprises a list of functional parameters and test methods inspired by the ISO 10993-18 standard, which can be used for the identification and evaluation of the physical, chemical, morphological, and topographical properties of materials in finished medical devices, all having a strong impact in terms of verification and validation of the target performance characteristics.

The results of the tests reported in Table 1 (morphological and geometrical characteristics), Table 2 (physical and structural characteristics), and Table 3 (mechanical characteristics) demonstrate that the comparison of three production lots gave satisfactory results. All the measured characteristics appeared highly reproducible, with a very low level of batch-to-batch variability, thus, ensuring the robustness of the manufacturing process. With reference to the suitability of the device for the intended use, the following discussion will provide deeper insights into the set of functional specifications that were defined during the design controls to comply with end-use requirements.

The inner diameter of the device is a geometrical characteristic determined by the foreseen clinical use. The transversal dimension of the conduit must be large enough to accommodate the distal and proximal extremities of the severed nerve and to allow their fixation by suturing during surgery. The size of the nerve conduits currently produced is 1.5–2.0 mm inner diameter, which is optimized for repairing transected digital nerves [16]. To repair larger nerves located in other anatomical positions, larger devices will be required. Usually, marketed nerve conduits cover a range of discrete sizes from 1 mm to 10 mm.

The wall thickness can be a more critical characteristic. A relationship between the formation of neuromas in regenerated nerves tissues and the thickness of the conduit wall has been reported [17]. The wall-thickness of SILKBridge^®^ has been carefully designed considering the properties of the selected starting materials and the requirements in terms of mechanical performance, suturability, targeted degradation rate, and the permeability specifications [18]. In general, wall thickness values lower that 1 mm are highly suggested for nerve guide application, with 0.6 mm as the optimum target for the maximum value [19]. As shown in Table 1, the final device falls within this optimal range.

Linear density, i.e., the weight per unit length, allows for evaluating the amount of material implanted into the body. Silk, as any other non-autologous biomaterial, is likely to elicit a foreign body response at the site of implantation [7]. Moreover, it has been reported that silk-based devices implanted in soft tissues or with longer degradation times tend to induce a long-lasting inflammatory response than those with shorter degradation times or those located within hard tissues [8]. On this basis, it has been decided to keep the linear density as low as possible to reduce the impact of the device in terms of local reactions. The choice of a macro-porous texture of the textile braid for the central layer of the wall allowed for keeping the linear density of the final device largely below the threshold of 10 mg/cm, as defined in the design stage (Table 1).

The porosity of the wall of a nerve conduit is a key functional parameter. It must be optimized to maximize the influx of oxygen and nutrients from the interstitial fluid and to avoid the loss of neurotrophic factors secreted by Schwann cells at the distal stump. A wall porosity of about 80% or higher is considered ideal for peripheral nerve repair [17,18,19]. This target has been achieved with SILKBridge^®^, whose wall porosity higher than 80% results from the combination of the macro- and micro-porosity of the TEX and ES layers, respectively (Table 1). With reference to the pore size, this characteristic is entirely determined by the two ES layers and does not exceed the 5 μm threshold on average (data not shown). This allows for the permeation of small nutrient molecules, while preventing the migration of inflammatory cells and the infiltration of fibrous tissue inside the lumen of the conduit [18].

The physical and structural features of the device were explored by FTIR and DSC analyses (Table 2). Both the spectroscopic and thermal properties of silk fibroin-based materials have been extensively investigated [20,21]. The type, position, and intensity of the bands in the IR spectra and of the thermal transitions in the DSC thermograms not only reflect the chemical structure of the silk materials but also provide information about the molecular conformation taken by the silk fibroin chains in native and regenerated formats.

The aim of the FTIR analysis was to verify that the manufacturing process did not alter the chemical integrity of the constituent material, did not leave foreign matters or contaminants on the device, and allowed for achieving the desired degree of crystallinity of the regenerated electrospun layers. Loss of integrity and changes in the chemical and conformational structure of the constituent silk material may have a strong impact on the biodegradation rate of the device upon implantation. Moreover, the presence of contaminants may impact on the biocompatibility of the device. For the purpose of integrity and purity checks, the position and intensity of the IR bands found in the spectrum of the sample were compared with reference spectra, while the crystallinity index was calculated from the intensity ratio of the Amide III bands at 1260 cm^−1^ (crystalline phase) and 1230 cm^−1^ (amorphous phase) [15].

The major thermal events characteristics of silk fibroin fall in the 150–400 °C temperature range (glass transition and melting/degradation). As with FTIR, the aim of the DSC analysis was to verify the structural integrity of the constituent silk fibroin materials to avoid any impact on the performance of the device. The DSC analysis allowed us to also calculate the mass ratio between the electrospun and textile layers, which present distinct endothermic peaks [10]. This parameter is a very useful tool to further assess the robustness of the manufacturing process. The DSC and FTIR results listed in Table 2 allowed for confirming the structural integrity of the silk fibroin materials comprising the device.

Medical devices must be designed with an optimal set of mechanical properties fitting the requirements of the specific clinical need. Nerve conduits must provide a balanced combination of strength and elasticity to withstand clinical operation stresses, such as manipulation and suturing during implantation, to resist deformation caused by the biomechanical stresses generated in vivo, and to avoid channel collapse, since compression can result in damage to the growing axon [10,11]. To verify that the manufacturing process allows for reaching the desired mechanical characteristics, the devices were analyzed for determining the tensile, suture retention, and compression properties (Table 3).

To withstand physiological loads, the tensile properties of nerve conduits should at least approach the ones of natural nerves [18,22]. The device must be elastic enough to match the deformation of the natural nerve and strong enough to protect the growing axons from breaking. From the data reported in the literature, the stress and strain values of human nerves are about 7 MPa and 60%, respectively. Considering the geometry of the device, the stress value corresponds to a breaking load of about 15 N. As shown in Table 3, the SILKBridge^®^ nerve conduit largely met the target values.

The suture retention test is intended to verify whether the device withstands the mechanical stresses applied by the surgeon during implantation and later during in vivo functioning. Suture failure may cause dramatic consequences to the patients, due to the loss of stability of the implant, lack of support for tissue regeneration, and need to re-operate. No threshold values for suture retention strength of nerve conduits have been reported in literature. On the contrary, limits were imposed for vascular grafts, a much more demanding application in terms of anastomotic strength, which requires that grafts are capable to exceed 2 N as suture retention strength [23,24,25]. Thanks to the strength of the textile braid, which is the load-bearing element of the wall of the device, the target threshold of suture retention strength was achieved by the final device (Table 3).

Finally, nerve conduits must withstand the mechanical compression stresses of surrounding tissues until complete nerve regeneration, avoiding collapse that may hinder the healing and cause pain to the patient [18]. To set acceptable threshold values for the compression performance, reference was made to the limit pressure values reported for the human median nerve exposed to carpal tunnel pressure [26]. In healthy individuals, carpal tunnel pressures are typically well below 10 mmHg, corresponding to a stress 1.33 kPa. Considering the geometry of the SILKBridge^®^, this stress corresponds to a compression load of about 1.1 cN. The results listed in Table 3 indicate that the compression resistance threshold was easily achieved, and that the device can withstand compression stresses and remain open to allow for a smooth progression of the nerve regeneration.

The evaluation of the potential toxicological risks associated with leachable substances released by a medical device in the surrounding tissues is another important step for the identification and quantification of the biological hazards related to its use. The ISO 10993-12 standard provides provisions for the preparation of samples for analysis. The ISO 10993-18 standard specifies a framework for the identification of leachable substances and for the quantitative determination of their potential release in the human body. The ISO 10993-17 standard guides the manufacturer through the process of estimation and control of the toxicological risks associated with the medical device use. The chemical analysis addressed all the processing aids used along the manufacturing process. Their amount was determined by applying suitable laboratory extraction conditions and advanced analytical methods.

With reference to the mechanism by which the chemical compounds may be released into the tissues surrounding the implant area, a combination of contributions can play a role. Simple diffusion from the device to the tissue may occur in the first period after implantation, when the device is still intact. Afterwards, when the device starts to degrade, swelling and fragmentation of the polymer texture may open new ways for the chemical to leach outside. However, it must be considered that the release will never occur in a bursting way, but gradually over time and in a more physiologically compliant manner, so that the local load is diluted over time. This assumption is supported by the fact that SILKBridge^®^ is made of silk fibroin materials characterized by a slow rate of degradation, i.e., from months to years for regenerated electrospun fibers and native microfibers, respectively [12].

As shown in Table 4, the results of the toxicological evaluation have demonstrated that the leachable substances coming from the manufacturing process were under control and that the cleaning procedures were effective in removing the greatest part of the processing aids from the final device. The residual amounts still present were largely below the threshold for toxicological concern, also considering the worst-case scenarios taken into account during the toxicological evaluation, i.e., the possibility of multiple implants (up to 10, one device for each finger), the ten-fold increase of the acceptable value of the Margin of Safety, and the assumption that the total amount is released in one day in a bursting way.

The targeted toxicological analysis here reported has been complemented with an untargeted one, which allowed to identify possible leachable compounds beyond those used for manufacturing, to include also unpredictable contaminants of environmental source, including laboratory materials, packaging, etc. (data not shown). It is worth noting that also this additional approach did not reveal the presence of unexpected contaminants likely to pose a health risk. 

The evaluation of the toxicity profile of a degradable medical device such as SILKBridge^®^ cannot disregard the possible toxicity of the degradation products. This issue has been addressed in a previous study where the in vitro degradation profile and the cytotoxicity of the degradation products were reported [12]. The bacterial protease type XIV from *Streptomyces griseus* was used as hydrolytic agent at three different enzyme/substrate ratios and for incubation times as long as 91 days. Degradation of the device occurred by surface erosion. The mass spectrometry analysis of the degradation products showed that the silk fibroin polypeptides recovered in the incubation buffers were representative of the aminoacidic sequence of the fibroin light and heavy chains, indicating that virtually the entire sequence of the fibroin protein was degraded. More important, the incubation buffers containing the soluble degradation products were tested with human HEK293 cells and mouse neuroblastoma N2a cells to assess cytotoxicity. No detrimental effects on cell viability were observed, suggesting that the degradation products, consisting of amino acids and small peptides, did not show any toxic property and their more likely fate was to enter the metabolic pathways of the host tissue cells [7]. Therefore, it is possible to conclude that the SILKBridge^®^ nerve conduits will not elicit any toxic effect due to the constituent materials and/or to the manufacturing process.

Biocompatibility is an essential requirement for the medical devices and the large amount of scientific data on the safety of silk is not sufficient on a regulatory prospective to allow the use of silk-based device since the manufacturing process can change the characteristics of the raw material and have an impact on the biocompatibility of the finished device [8]. This perspective imposes that a comprehensive evaluation of the device biocompatibility is carried out to prevent any possible adverse effect for the patient’s health. Different parts of the ISO 10993 standard provide a framework for the evaluation of the biological safety of the device. A plan has been designed and a set of tests, including cytotoxicity, delayed hypersensitivity, intracutaneous reactivity, pyrogen test, LAL test, acute systemic toxicity, and genotoxicity has been carried out. The overall results of the biological response allowed confirming that SILKBridge^®^ is fully biocompatible (Table 5). This is in good agreement with the numerous data reported in the scientific literature about the biocompatibility of silk materials [8].

Finally, the evaluation of a medical device can’t exempt from carrying out performance tests to verify that the product achieve it’s intended use. In a previously published article [11] the implantation of the SILKBridge^®^ in the median nerve of rats was discussed to demonstrate the regeneration of myelinated fibers along the conduit filling the gap previously created and leading to an effective morphological and functional recovery of the median nerve, like that observed with the reference autograft nerve reconstruction.

Altogether, the results reported here, and others previously published [10,11,12] represent an important achievement towards the implementation of a clinical study aimed at investigating the safety and efficacy of the SILKBridge^®^ nerve conduit. The device has demonstrated an optimized balance of biomechanical and biological properties; it is intended as an “off-the-shelf” product ready to be used in the operating room as it is, without the need of adding neurotrophic and/or angiogenic factors or cells. The encouraging on-bench and pre-clinical results allowed us to proceed quickly towards the submission of a first in-human clinical study aimed at evaluating the reconstruction of digital nerve defects in humans (ClinicalTrials.gov identifier: NCT03673449). The study has already started at the Department of Plastic Surgery and Hand Surgery of the University Hospital of Zurich. Four out of 15 patients have been enrolled so far and implanted with SILKBridge^®^ nerve conduit to repair a digital nerve gap, with very satisfactory outcome after one year in terms of functional recovery.

## 5. Conclusions

The path from silk-based starting materials (cocoons, yarns) to the production of a medical device and to its use in humans is long and tortuous. Patient safety and health are primary goals that must not be jeopardized in any kind of approach aimed at solving clinical problems. Of course, it is not surprising that regulatory agencies have put in place a set of very thorough and detailed directives to objectively assess all the risks associated with the use of implantable medical device.

As shown in this study, transforming a silk material into a medical device and taking it all the way to clinical application requires a network of expertise ranging from polymer science, chemistry, toxicology, biomedical engineering, medicine, biology, regulatory, and quality. It is also necessary to demonstrate that all manufacturing processes leading to the final device are scalable from laboratory or pilot scale to industrial scale. Finally, the support of clinicians is also of great importance from the beginning, with the identification of the clinical need to be solved, until the end, to evaluate the clinical results and to support product marketing.

## Figures and Tables

**Table 1:** On-bench testing results (mean ± Std. Dev.): morphological and geometrical characteristics (*n* = 5).

	Batch A	Batch B	Batch C	*p* Value ^1^
Internal diameter (mm)	1.78 ± 0.10	1.76 ± 0.04	1.82 ± 0.07	>0.05
Wall thickness (mm)	0.43 ± 0.05	0.47 ± 0.04	0.47 ± 0.04	>0.05
Linear density (mg/cm)	5.7 ± 0.4	5.8 ± 0.2	6.2 ± 0.3	>0.05
Porosity (%)	86 ± 2	87 ± 2	86 ± 2	>0.05

^1^ One-way ANOVA (confidence interval of the mean 95%). All data followed a normal distribution.

**Table 2:** On-bench testing results (mean ± Std. Dev.): physical and structural characteristics (*n* = 5).

	Batch A	Batch B	Batch C	*p* Value ^1^
Degree of crystallinity	0.66 ± 0.03	0.66 ± 0.03	0.67 ± 0.01	>0.05
Thermal properties:				
T_ES_ (°C)	291 ± 1	291 ± 2	290 ± 1	>0.05
T_TEX_ (°C)	321 ± 1	322 ± 1	321 ± 2	>0.05
ΔH_ES/TEX_ (J/g)	411 ± 21	402 ± 32	410 ± 31	>0.05
ES:TEX ratio (%)	53 ± 1	53 ± 3	54 ± 2	>0.05

^1^ One-way ANOVA (confidence interval of the mean 95%). All data followed a normal distribution.

**Table 3:** On-bench testing results (mean ± Std. Dev.): mechanical characteristics (*n* = 5).

	Batch A	Batch B	Batch C	*p* Value ^1^
Suture retention (N)	4.6 ± 0.4	4.5 ± 0.8	4.7 ± 0.8	>0.05
Compression strength:				
Load at 20% strain (cN)	4.6 ± 1.2	4.2 ± 1.0	4.6 ± 1.0	>0.05
Load at 40% strain (cN)	9.2 ± 1.6	8.6 ± 1.9	9.7 ± 1.7	>0.05
Modulus (kPa)	93 ± 12	84 ± 17	97 ± 14	>0.05
Tensile strength:				
Breaking load (N)	24.8 ± 0.5	23.3 ± 0.9	23.1 ±1.8	>0.05
Elongation at break (%)	59.5 ± 2.7	60.5 ± 6.5	61.1 ± 3.6	>0.05

^1^ One-way ANOVA (confidence interval of the mean 95%). All data followed a normal distribution.

**Table 4:** Chemical and toxicological analysis of leachable processing aids.

		LiBr		EtOH	MeOH	EMIMAc	FA
		Li^+^	Br^−^				
1	Concentration (mg/kg)	4.0	324	2.7	1	4.1	200
2	Total Released Amount (μg/device) ^1^	0.1	8.0	0.07	0.03	0.1	5.0
3	Potential Patient Daily Intake (PPDI; μg/day)	1.0	80	0.7	0.3	1.0	50
4	Tolerable Exposure (TE; μg/day)	250	1750	50,000	9000	10 ^2^	35,400
5	Margin of Safety (MoS)	0.25 × 10^3^	22	71 × 10^3^	30 × 10^3^	10	0.71 × 10^3^

^1^ An average weight of 25 mg has been considered for the device. ^2^ The TTC (threshold of toxicological concern) is used for EMIMAc instead of TE.

**Table 5:** Results of the biological safety evaluation.

Test	Evaluation	Final Score
Cytotoxicity	After 24 h of contact with the test sample extracts, discrete intracytoplasmic granules, no cell lysis, and no reduction of cell growth were observed (reactivity grade 0). The cell viability reduction was 4%.	Not cytotoxic
Delayed hypersensitivity	No abnormalities were observed in treated and control animals during the challenge phase (grade 0).	Not sensitizing
Intracutaneous reactivity	No abnormalities were observed immediately after injection. With polar extract all treated and control sites did not show signs of erythema, eschar or edema. With apolar extract, all treated and control sites showed a slight erythema, no eschar or edema. The Primary irritation index was 0.	Not irritating
Pyrogen test	The summed temperature rise of rabbits was +0.3 °C and the summed response did not exceed 2.8 °C. Only an individual temperature rise of one rabbit was higher than 0.5 °C and the summed temperature rise was lower than 3.3 °C.	Not pyrogenic
LAL test	The test sample solution had an endotoxin content <20 EU/device.	Not endotoxic
Acute systemic toxicity	None of the treated and control animals showed toxic signs, symptoms, mortality, or weight loss.	Not toxic
Genotoxicity	Bacterial Reverse Mutation Test: no toxic effects of the extracts were noted in any of the tester strains used; no biologically relevant increases in revertant colony numbers of any of the tester strains were observed following treatment with extracts. Mouse Lymphoma Test: no growth inhibition was observed for the polar and lipophilic extracts; no biologically relevant increase of mutants was found for the polar and lipophilic; the global evaluation factor (GEF) was not exceeded by the induced mutant frequency at any concentration; no dose-response relationship was observed.	Not mutagenic

## Data Availability

The datasets generated for this study are available on reasonable request to the corresponding author.
